# Do Hybrid Trees Inherit Invasive Characteristics? Fruits of *Corymbia torelliana* X *C*. *citriodora* Hybrids and Potential for Seed Dispersal by Bees

**DOI:** 10.1371/journal.pone.0138868

**Published:** 2015-09-29

**Authors:** Helen Margaret Wallace, Sara Diana Leonhardt

**Affiliations:** 1 Genecology Research Centre, Faculty of Science, Health, Education and Engineering, University of the Sunshine Coast, Maroochydore DC 4558, Australia; 2 Department of Animal Ecology and Tropical Biology, University of Würzburg, 97074, Würzburg, Germany; University of A Coruña, SPAIN

## Abstract

Tree invasions have substantial impacts on biodiversity and ecosystem functioning, and trees that are dispersed by animals are more likely to become invasive. In addition, hybridisation between plants is well documented as a source of new weeds, as hybrids gain new characteristics that allow them to become invasive. *Corymbia torelliana* is an invasive tree with an unusual animal dispersal mechanism: seed dispersal by stingless bees, that hybridizes readily with other species. We examined hybrids between *C*. *torelliana* and *C*. *citriodora* subsp. *citriodora* to determine whether hybrids have inherited the seed dispersal characteristics of *C*. *torelliana* that allow bee dispersal. Some hybrid fruits displayed the characteristic hollowness, resin production and resin chemistry associated with seed dispersal by bees. However, we did not observe bees foraging on any hybrid fruits until they had been damaged. We conclude that *C*. *torelliana* and *C*. *citriodora* subsp. *citriodora* hybrids can inherit some fruit characters that are associated with dispersal by bees, but we did not find a hybrid with the complete set of characters that would enable bee dispersal. However, around 20,000 hybrids have been planted in Australia, and ongoing monitoring is necessary to identify any hybrids that may become invasive.

## Introduction

Global interest in invasive trees is increasing and invasive trees have recently been recognized as having substantial impacts on biodiversity and ecosystem functioning [1]. There are many factors that may contribute to trees becoming invasive, and some critical factors include tree dispersal mechanisms, hybridisation and escape from plantations. Tree species dispersed by animals are disproportionality more likely to become invasive and long-distance seed dispersal, often a feature of animal dispersal, can play a crucial role in invasions [2, 3]. Invasion may also be facilitated by hybridisation [4]. Hybridisation is an important evolutionary process for plants that allows them to evolve rapidly and can confer new characteristics that enable plants to proliferate, persist and invade [5]. Hybridisation between domesticated plants and wild relatives is well documented as a source of new weeds [4, 6]. Lastly, tree invasions are often driven by tree species escaping from cultivation or plantations [7]. Thus management strategies for tree plantations need to account for the possibility of seed dispersal into surrounding areas and subsequent invasions [7].

*Corymbia torelliana* is an invasive tree species with an unusual animal dispersal mechanism [8, 9]. The tree is dispersed by stingless bees foraging for resin inside the fruit [8–11]. Stingless bees collect plant resins for nest building and defence [12, 13]. Seeds taken up by resin collecting bees may be dispersed long distances (over 300 m), and the tree is invasive in areas outside of its natural range where it co-occurs with stingless bees [8, 10]. *C*. *torelliana* is the only *Corymbia* species to be dispersed by bees and to be invasive. Other members of the genus are not invasive and are dispersed very short distances through barochory. Furthermore *C*. *torelliana* is invasive only in areas where it co-occurs with stingless bees [8, 10]. This suggests that this unique dispersal mechanism contributes greatly to making *C*. *torelliana* invasive. Moreover, *C*. *torelliana* (section Cadagaria) hybridizes readily with three closely related species in the spotted gum group, (section Politaria) *Corymbia citriodora* subsp. *citriodora*, *C*. *citriodora* subsp. *variegata*, *C*. *henryi* and *C*. *maculata* [14–17]. In addition, *C*. *torelliana* is able to form hybrids with more distantly related *Corymbia* species [15]. *Corymbia* species and their hybrids are of increasing interest for forestry plantations globally due to their fast growth and resistance to disease. Over 20,000 ha of plantations have been established across Australia since the late 1990s primarily using *Corymbia citriodora* subsp. *variegata* [18, 19]. The *C*. *torelliana* hybrids may pose a risk to native forests if they have the same fruit characteristics that would enable long distance dispersal by bees and could allow the plantation trees to become an invasive weed [18]. Hybrids with *C*. *torelliana* could inherit characteristics that allow them to be dispersed by bees and become invasive. However, *C*. *torelliana* has a fruit structure that is unique in *Corymbia* and enables seed dispersal by bees. The central column collapses and the valves retract resulting in a completely hollow fruit [20, 21]. As a result, the open fruits of *C*. *torelliana* are hollow spherical structures, which permit entry of the bees [8, 10]. To our best knowledge, the hybrid fruits have as yet not been examined to determine their structure and their attractiveness to bees.

The chemical composition of the fruit resin further plays a critical role in the relationship with the seed-dispersing bees, as bees use a complex set of olfactory cues to find the resin [22]. Stingless bees incorporate resin compounds into their cuticle and compounds also found in *C*. *torelliana* fruit resin have been discovered in seven species of Australian stingless bees [23–25]. Bees with resin-derived compounds (including those of *C*. *torelliana*) are better protected against predators, e.g. ants [26] Moreover, *C*. *torelliana* resin is more effective as an antimicrobial agent than three other tree resins frequently collected by stingless bees [27], suggesting an important role of this resin in protecting stingless bee colonies against pathogenic microbes. Therefore, bees may even benefit from a spread of invasive *C*. *torelliana* trees. However, some beekeepers claim that *C*. *torelliana* resin may harm stingless bee colonies e.g. by causing colonies to collapse [28] although no evidence of this has been found in areas where they co-occur [9]. Whether the chemical composition of resin and subsequently the chemical ecology of stingless bees collecting resin is affected by hybridisation is unknown.

The aim of this study was to investigate whether hybrids of *C*. *torelliana* have the same characteristics of the parent tree that would enable seed dispersal by bees, and may facilitate invasion. We specifically addressed the following questions: (1) what are the external and internal dimensions of hybrid fruits compared with *C*. *torelliana* (2) how frequently do hybrids display the critical characteristics of resin presented in the fruit and central column collapse associated with the *C*. *torelliana* seed dispersal syndrome (3) what hybrid fruits if any are attractive to bees? (4) what is the chemical profile of the resin from fruits of hybrids compared with *C*. *torelliana*? We expected to find some hybrids with similarities to *C*. *torelliana* that may enable long distance dispersal by bees and thus may facilitate invasion by hybrids.

## Materials and Methods

### Study site

**Fig 1:**
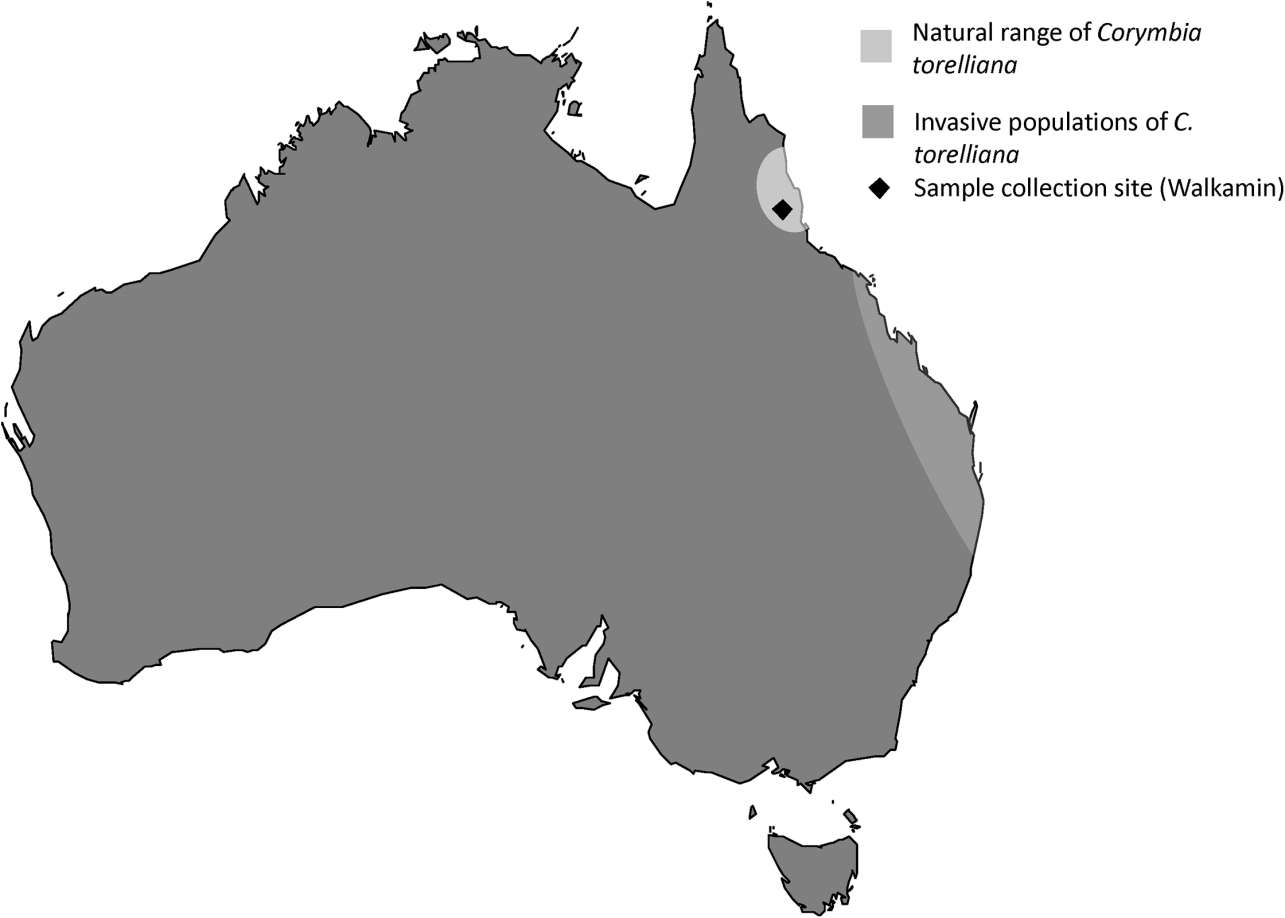
Map of Australia showing natural range of *Corymbia torelliana*, the study site (Walkamin, QLD) and the location of invasive populations.

We examined a windbreak planting of *C*. *torelliana*, *C*. *citriodora* subsp. *citriodora* and spontaneous hybrids of *C*. *torelliana* and *C*. *citriodora* subsp. *citriodora* on private land near Walkamin, North Qld, Australia (17° 8' 5" S, 145° 25' 41" E) (Fig 1). The owner of the land gave permission to conduct the study on this site. *Corymbia* species form hybrids across taxonomic groups and wide crosses between species from subgenera *Blakella* and *Corymbia* are known from natural populations [17] and controlled pollination studies [14, 16]. There were 97 trees of *C*. *torelliana*, *C*. *citriodora* and hybrids at the site. The parent species have very different tree form, bark and leaf characteristics. *C*. *citriodora* subsp. *citriodora* has pale whitish, pink or grey bark, with narrow lanceolate to lanceolate leaves with a distinctly lemon odour from the aldehyde citronellal contained in the leaves [17]. In contrast *C*. *torelliana* is distinctly different from all other known *Corymbia* species, with a tessellated stocking on the lower trunk and grey green to whitish green bark above shedding in thin sheets. The juvenile leaves of *C*. *torelliana* are peltate and setose and adult leaves are often poorly developed on mature *C*. *torelliana* trees [17]. The occurrence of *C*. *torelliana* hybrids with *C*. *citriodora* subsp. *citriodora* and other spotted gums and their morphological features have been widely reported in the literature [14–17]. Hybrids may have intermediate tree form, bark characteristics and leaf shapes or may show unusual combinations of parent features. Hybrids are often strikingly different from the parent species in windbreak plantings when planted alongside the parent species. We assessed morphological characteristics such as tree form, bark colour and texture, leaf shape and smell as described in [17] to determine the identity of all trees at the site as either pure species or hybrids. We examined all trees to determine whether they had fruits, and if so whether they were mature.

### Fruit dimensions

Where fruits were mature, we assessed fruit dimensions of *C*. *torelliana*, *C*. *citriodora* subsp. *citriodora* and hybrids of the two species. We observed 12 trees of *C*. *torelliana*, 10 trees of *C*. *citriodora* subsp. *citriodora* and 37 hybrid trees with mature fruits at the site during the study. We measured the fruit dimensions of 10 capsules per tree in terms of external length, external width, external rim diameter and internal rim diameter.

### Fruit characteristics

We examined the structure of hybrid fruits to determine whether they had the *C*. *torelliana* characteristics that may influence seed dispersal by bees. We sectioned five fruits from each of 16 hybrid trees (80 hybrid fruits) transversely and determined whether resin was present, and whether the central column had collapsed. Fruits were assigned a score of 1, 2 or 3 where 1 was little resin, 2 was some resin and 3 was copious resin. Column collapse was assigned a rank of 1, 2 or 3 where 1 was column intact, 2 was column partially collapsed and 3 was column fully collapsed. Five fruits of each of 3 *C*. *torelliana* and *C*. *citriodora* subsp. *citriodora* were sectioned to assist in comparing the hybrids with the parent species.

### Bee behaviour

*C*. *torelliana*, *C*. *citriodora* subsp. *citriodora* and hybrids were examined for 10 days over 3 fruiting seasons between 2002 and 2005 to determine whether bees foraged for resin on the fruits and whether bees subsequently dispersed seeds. All observations were carried out between 09.30 and 0.4.30 during sunny weather conducive to bee activity. Each tree was initially examined to determine the maturity of the fruits. *Corymbia* species typically have clusters of fruits that protrude from the canopy of the tree [17].

The fruit clusters with up to 50 fruits were examined for signs of opening, e.g. mottled green/brown in colour and opening valves. Where fruits were opening, they were observed for 5 minutes for bee activity using Zeiss 8 X 30 binoculars. For each tree observed, the number of bees foraging on the cluster of fruit during the 5 minute observation period was recorded. Fruits were observed to determine whether bees landed on the outside and entered the fruits. If bees were observed leaving the fruits, observations were made on whether they carried resin, chaff or seeds. One cluster of fruits per tree was observed for 5 minutes for each tree for each day. Observations on all trees were repeated every 2–4 days over 2–3 weeks in 2004 and 2005. In preliminary observations, we found that bees foraged on hybrid fruits that had been chewed and discarded by red tailed black cockatoos. Where trees had fruits that had been discarded on the ground, we conducted further observations of bee activity on the ground for a 5 minute period. We observed all fruits on the ground within a 3m× 3m square to determine whether bees were attracted to fruits.

### Resin chemistry

To test whether the chemical composition of resin in fruits of *C*. *torelliana×C*. *citriodora* hybrids and *C*. *torelliana* differed qualitatively (presence/absence of compounds) and/or quantitatively (different proportions of compounds), resin was analysed using gas chromatography (GC) and mass spectrometry (MS) as described in [23]. GC separates compounds according to their molecular weight and chemical structure, and MS breaks them down into fragments that are typical for specific compounds (classes). These fragments can then be used for identification and comparison with references.

We analysed resin from three *C*. *torelliana* and three hybrid trees. Resin of *C*. *torelliana* has previously been analysed and shows little variation between trees or populations (see [24] [29]), which is why we considered three trees sufficient as a reference to which we could compare resin from hybrids. However, although we obtained capsules from overall 16 hybrids, only three of them produced fruits with sufficient amounts of resin for chemical analyses. Where available, resin of 3–5 fruits (between 5–30 μl resin/ fruit) per tree was extracted with a clean knife and forceps and transferred to 2.5 ml glass vials containing hexane (p.a. grade, Sigma-Aldrich, Munich, Germany).

For characterization of compounds we used a Hewlett Packard HP 6890 Series GC System coupled to a Hewlett Packard HP 5973 Mass Selective Detector (Agilent Technologies, Böblingen, Germany). The GC was equipped with a J & W, DB-5 fused silica capillary column (30m x 0.25 mm ID; df = 0.25 μm; J & W, Folsom, CA, USA). Temperature was programmed from 60°C to 300°C with 5°C/min heating rate and held for 10 min at 300°C. We used helium as carrier gas (constant flow of 1 ml/min). Injection was carried out at 250°C in the splitless mode for 1 min. The electron impact mass spectra (EI-MS) were recorded at 70 eV and 230°C. Windows version of the ChemStation software package (Agilent Technologies, Böblingen, Germany) was used for data acquisition and analysis.

Chemical compounds found in hexane extracts of resin were characterized by their mass spectra and their retention times. We regarded peaks with identical mass spectra and retention times as the same compound. Three commercially available mass spectra libraries (Wiley 275, NIST 98 and Adams EO library 2205) were used to determine compound classes with regard to their mass spectra and retention indices (Kovats Index). Moreover, all alkanes were confirmed by synthetic standards (Sigma-Aldrich, Munich, Germany). Aldehydes, alcohols and esters as well as terpenes were tentatively identified by comparison of the obtained mass spectra with mass spectra and retention indices in libraries. Comparisons with synthetic standards (Sigma-Aldrich, Munich, Germany) were performed if standards were available. Data of terpenes were further compared with those of dipterocarp and pine tree resins that typically comprise di-, sesqui- and triterpenes [30].

### Statistical analysis

All data on fruit dimensions were analysed with a nested ANOVA with taxa and tree (nested within taxa) as factors followed by Tukey’s HSD test where significant differences between taxa were detected. Prior to the statistical analysis of chemical compounds, trace compounds for which mass spectra could not be interpreted as well as compounds which accounted for less than 0.5% of the total peak area in all samples were removed from the dataset (if a compound accounted for more than 0.5% in one samples, it was included in the analysis although it may have accounted for less than 0.5% in other samples). The analysis was based on a total of 97 compounds. These compounds were quantified as proportions by dividing the peak area of each compound by the total area of all sample peaks included in the analysis.

We examined the chemical variation among resins from *C*. *torelliana* and hybrid trees using (a) all compounds, and (b) only volatile compounds. We considered compounds volatile when they eluted from the GC column prior to 23 min sample run time. In doing so, we may have included few non-volatile polar compounds, but the dataset will be largely confined to volatile compounds. We then analysed chemical variation between tree resins by a multivariate matrix permutation test based on the Bray-Curtis distances between compounds using the ADONIS function in the VEGAN package in R. To identify compounds contributing to the variation observed between resins of *C*. *torelliana* and hybrids, a principal covariate analysis (PCoA) also based on Bray-Curtis distances was performed. Finally, a hierarchical cluster analysis based on the Bray-Curtis distances between compounds was used to produce figures. All statistical analyses were performed in R [31].

## Results

### Fruit dimensions and characteristics

**Fig 2:**
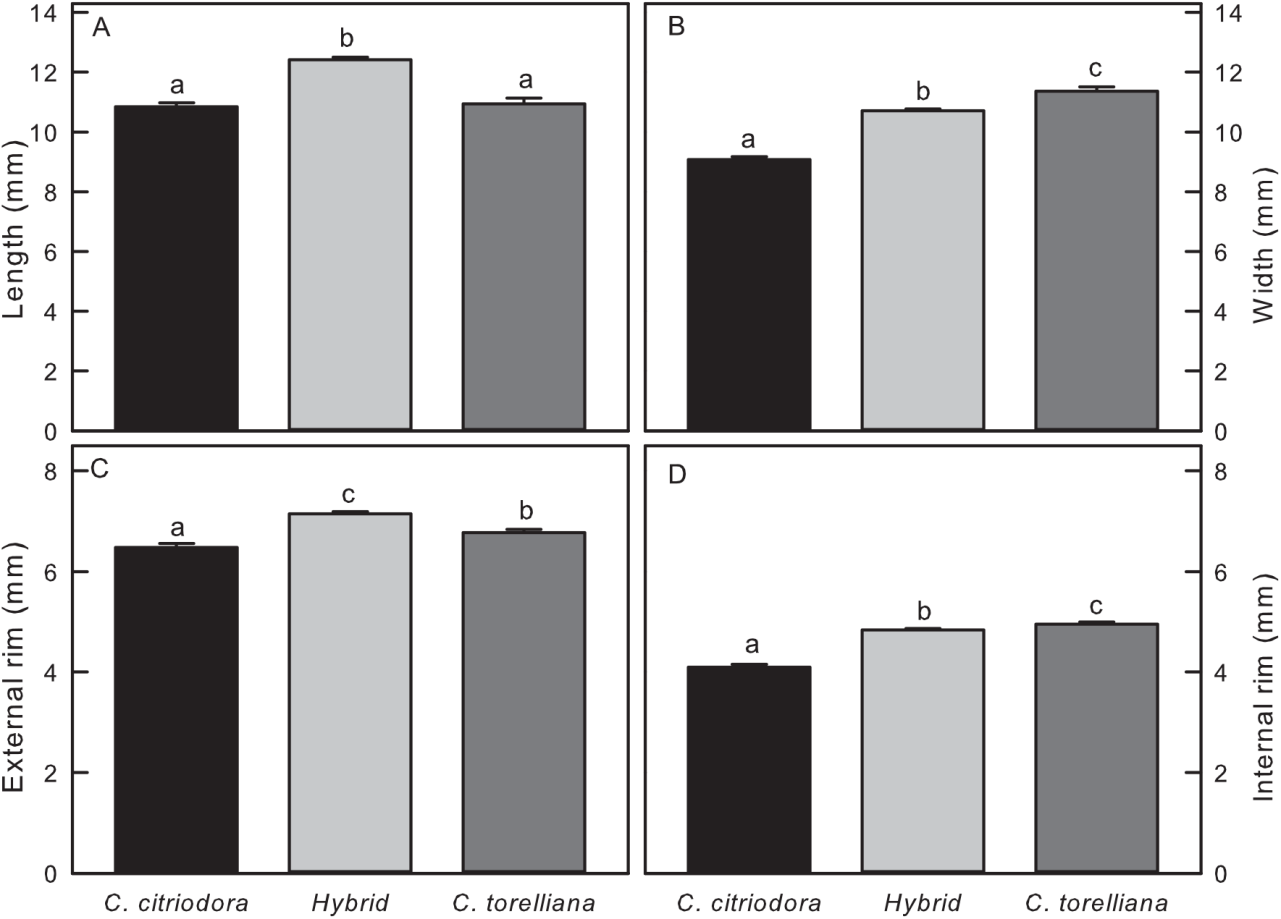
Fruit dimensions of *Corymbia torelliana*, *C*. *citriodora* subsp. *citriodora* and hybrids: A) Length, B) Width, C) External Rim, D) Internal Rim. Means and standard errors are presented, means with different letters are significantly different (Tukey’s HSD, P<0.05)

There were significant differences between the three taxa in all fruit dimensions sampled. Both *C*. *torelliana* and *C*. *citriodora* subsp. *citriodora* were significantly shorter than hybrids (F = 165.23, *P*<0.0001, Fig 2A). *C*. *torelliana* was significantly wider than *C*. *citriodora* subsp. *citriodora* with hybrids showing intermediate width (F = 383.64, *P*<0.0001, Fig 2B). Hybrids had a significantly larger external rim than both species (F = 81.59, *P*<0.0001, Fig 2C). The internal rim was widest for *C*. *torelliana*, smallest for *C*. *citriodora* subsp. *citriodora* and hybrids were intermediate (F = 272.46, *P*<0.0001, Fig 2D). Within the three taxa there were significant differences between individual trees for height, width, external rim and internal rim (F = 27.27, F = 32.16, F = 24.44, F = 21.49 respectively, P<0.0001 in all cases).

**Fig 3:**
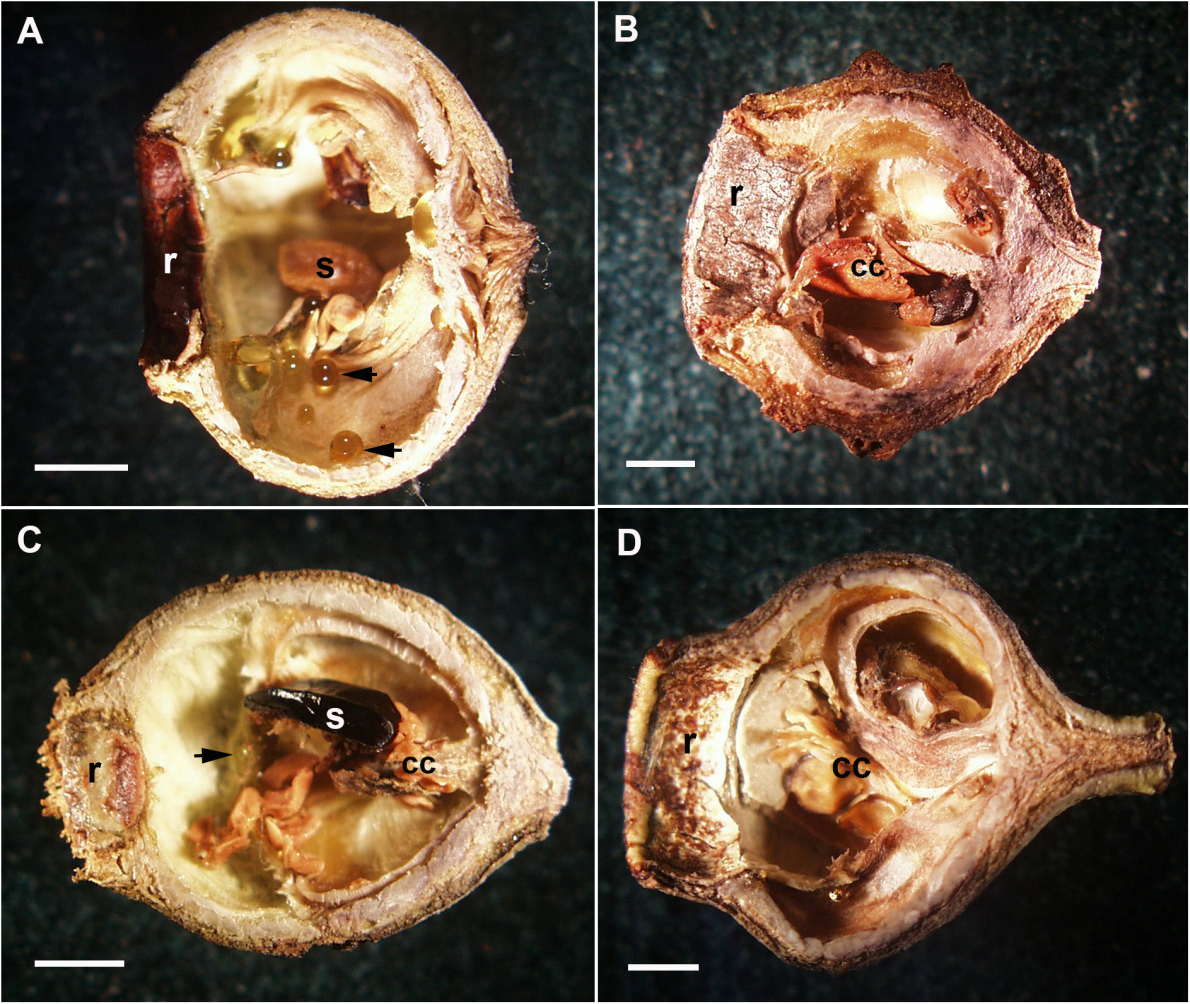
Fruit structure of *Corymbia torelliana*, *Corymbia citriodora* subsp. *citriodora* and *C*. *torelliana* ×*C*. *citriodora* subsp. *citriodora* hybrids. All bars = 2mm A. *C*. *torelliana* fruit showing hollow structure with copious resin (black arrows). Note the width of the internal rim (r) B. *C*. *citriodora* subsp. *citriodora* showing central column (cc) and small internal dimensions and small woody rim (r). C. *C*. *torelliana* ×*C*. *citriodora* subsp. *citriodora* hybrids partial central column (cc) and large seed (s) Note the small rim (r) compared with A, and the presence of small amounts of resin (black arrow). D. *C*. *torelliana* ×*C*. *citriodora* subsp. *citriodora* hybrid showing partial central column (cc). note the large rim (r).

All fruits had resin present, although 6 of the 16 hybrid fruits sectioned had very little (characteristic of *C*. *citriodora*, Table 1, Fig 3B). Only one hybrid displayed the copious and viscous resin characteristic of *C*. *torelliana* (Fig 3A). Similarly, only four of the 16 hybrids examined showed complete central column collapse, characteristic of *C*. *torelliana*, and 5 showed intermediate column collapse (Figs 3C and 2D; Table 1). Seeds in some hybrid capsules were noticeably larger than *C*. *torelliana* (Fig 3C).

**Table 1:** Number of *Corymbia torelliana* × *C*. *citriodora* subsp. *citriodora* hybrid trees displaying *C*. *citriodora* subsp. *citriodora*, intermediate or *C*. *torelliana* fruit characteristics. Resin was present in small quantities in *C*. *citriodora* and plentiful in *C*. *torelliana*. The central column structure was intact in *C*. *citriodora* and collapsed in *C*. *torelliana* (see Fig 2). There were 16 hybrids assessed in total.

Fruit characteristics	Percentage of hybrids with *C*. *citriodora* fruit character	Percentage of hybrids with intermediate fruit characters	Percentage of hybrids with *C*. *torelliana* characters
Resin content	37.5	56.25	6.25
Column structure (intact or collapsed)	43.75	31.25	25

### Bee behaviour

Bees did not visit fruits on trees of any hybrids or of *C*. *citriodora* subsp. *citriodora*. In contrast, at the same site, there were on average 2.75 bee visits per 5 minutes to the *C*. *torelliana* fruits (Table 2). Bees visiting the *C*. *torelliana* fruits were frequently observed entering capsules and carrying resin loads in their corbiculae when leaving capsules. Bees frequently visited broken fruits of hybrids on the ground to collect resin from the wound tissue (Table 2). Bees were observed carrying resin and chaff from these fruits, but seed dispersal by stingless bees from these fruits was not observed.

**Table 2:** Attractiveness of *C*. *citriodora* subsp. *citriodora*, *C*. *torelliana* and *Corymbia torelliana* × *C*. *citriodora* subsp. *citriodora* hybrids to stingless bees. Mean (standard error) bee visits per 5 minute observation period is given. No. of trees = Number of trees for this taxa with mature capsules observed, No. of obs = total number of observations for this taxa.

Species	Location of fruits	Mean bee visits (se)	No. of trees	No. of obs.
*C*. *citriodora* subsp. *citriodora*	Tree	0	6	45
*C*. *torelliana*	Tree	2.75 (0.48)	4	32
Hybrids	Tree	0	25	167
Hybrids	Ground	3.24 (0.74)	7	29

### Resin chemistry

**Fig 4:**
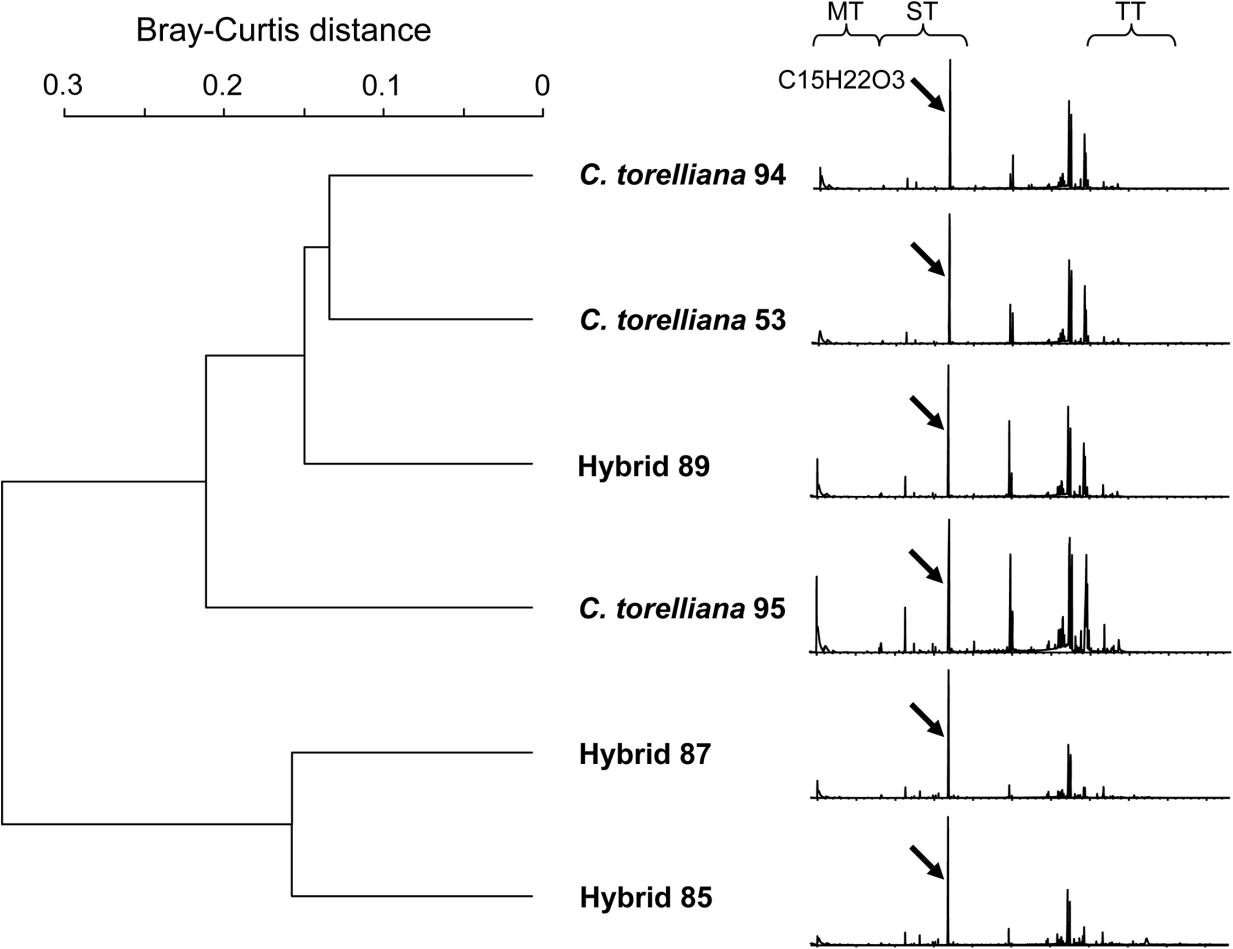
Dendrogram (derived from hierarchical cluster analysis based on Bray-Curtis distances between compounds) of chemical distances between resin of *Corymbia torelliana* and *C*. *torelliana* × *C*. *citriodora* subsp. *citriodora* hybrids (hybrid 85, 87 and 89) with chromatogram of each sample. Ranges where monoterpenes (MT), sesquiterpenes (ST) and non-volatile compounds, such as triterpenes (TT), typically dilute in our method are indicated by brackets. Arrows mark a flavonoid (C15H22O3) characteristically found in resin of *C*. *torelliana* fruits.

Resins of three *C*. *torelliana* trees and three hybrids differed significantly in their chemical composition (Adonis, all compounds: *R* ^*2*^ = 0.43, *P* < 0.001, Fig 4), as resins of hybrid 85 and hybrid 87 were chemically more distant from *C*. *torelliana* resin than resin of hybrid 89 (Fig 4). Differences were both qualitative and quantitative and could be attributed to several mono- and sesquiterpenes as well as to several unidentified non-volatile compounds (Table 3). However, when only volatile compounds were considered, resins from *C*. *torelliana* and hybrids did not significantly differ in their chemical composition (Adonis: *R* ^*2*^ = 0.35, *p* = 0.10).

**Table 3:** Percentages [± SD] of chemical compounds found in resin of fruits of three *Corymbia torelliana* and three *C*. *torelliana* -× *C*. *citriodora* subsp. *citriodora* hybrids, listed according to major ion (MI), Kovats Index (KI) and retention time (RT). PC1 and PC2 give loadings of 1st and 2nd axis derived from principal covariate analysis (PCoA), p-value (p) indicates whether a compound explains a significant proportion of the variance (. p < 0.1, * p < 0.05, ** p < 0.01).

Nr.	MI	Compound	KI	RT	*C*. *torelliana*	Hybrids	PC1	PC2	*P*	
1	136	Tricyclene	921	5.0	1.8 ± 1.5	2.3 ± 1	-0.07	1.00	0.43	
2	136	*α*-Pinene	932	5.1	9.4 ± 1.9	9.2 ± 0.5	-0.01	-1.00	0.03	*
3	136	monoterpene		5.9	0.1 ± 0	0.1 ± 0.1	-0.84	0.55	0.26	
4	136	*β*-Pinene	974	6.0	2.1 ± 1	1 ± 0.1	-0.33	-0.95	0.20	
5	136	monoterpene		6.3	0.6 ± 0.6	0.8 ± 0.1	0.31	0.95	0.87	
6	136	Limonene	1024	7.1	0.1 ± 0	0.1 ± 0	-0.23	-0.97	0.97	
7	136	monoterpene		7.2	0.1 ± 0.1	0 ± 0	-0.18	0.98	0.01	**
8	136	monoterpene		7.2	0.3 ± 0.2	0.2 ± 0.2	-0.13	-0.99	0.08	.
9	136	monoterpene		7.8	0 ± 0	0.1 ± 0.1	0.99	0.10	0.05	*
10	136	monoterpene		7.9	0 ± 0	0.3 ± 0.3	0.99	0.15	0.01	**
11	136	monoterpene		8.9	0.2 ± 0.1	0.1 ± 0.1	-0.17	-0.99	0.39	
12	136	monoterpene		11.5	0.1 ± 0	0.1 ± 0	0.05	-1.00	0.36	
13	170	unknown		11.6	0.1 ± 0.1	0.3 ± 0.2	0.81	-0.58	0.46	
14	164	Acetic acid, 2-phenylethyl ester		13.0	0.1 ± 0.2	0.1 ± 0.2	-0.41	0.91	0.14	
15	-	unknown		13.2	1 ± 0.1	1.2 ± 0.3	0.67	0.74	0.39	
16	184	Tridecane	1300	14.2	0 ± 0	0.1 ± 0.1	0.98	-0.20	0.44	
17	204	sesquiterpene		15.5	0.1 ± 0	0 ± 0	-0.92	-0.38	0.14	
18	204	sesquiterpene		16.1	0 ± 0	0.1 ± 0	0.99	0.17	0.06	.
19	204	*α*-Copaene	1374	16.3	1.5 ± 0.4	2.2 ± 0.1	0.36	0.93	0.24	
20	204	sesquiterpene		17.1	0 ± 0	0.2 ± 0.1	0.99	0.13	0.02	*
21	204	E-Caryophyllene	1417	17.5	0.5 ± 0.2	0.4 ± 0.1	-0.38	-0.93	0.20	
22	204	cis-Muurola-3,5-diene	1448	18.2	0.1 ± 0	0.9 ± 0.8	0.98	0.20	0.01	**
23	204	sesquiterpene		18.3	0.1 ± 0	0.1 ± 0	-0.42	-0.91	0.23	
24	204	sesquiterpene		19.2	0 ± 0	0.1 ± 0.1	0.99	-0.11	0.02	*
25	204	sesquiterpene		19.3	0.1 ± 0	0.1 ± 0	0.85	0.52	0.39	
26	204	sesquiterpene		19.3	0 ± 0	0.1 ± 0	0.96	0.28	0.27	
27	204	sesquiterpene		19.8	0.2 ± 0	0.4 ± 0.1	0.76	0.65	0.03	*
28	202	trans-Calamene	1521	19.9	0 ± 0	0.3 ± 0.2	0.97	0.25	0.05	*
29	204	sesquiterpene		20.2	0.1 ± 0.1	0.4 ± 0.2	0.84	0.55	0.03	*
30	222	Elemol	1548	20.6	0 ± 0	0.5 ± 0.5	0.99	0.14	0.01	**
31	250	Polar substance (C15H22O3)		21.8	18.1 ± 2.8	25.4 ± 7	0.84	-0.55	0.10	.
32	248	Hillone	1607	21.9	0.3 ± 0.3	0.5 ± 0.2	0.54	-0.84	0.11	
33	-	unknown		22.0	0.1 ± 0.1	0.2 ± 0.1	0.47	-0.88	0.10	.
34	-	unknown		22.2	0.1 ± 0	0.1 ± 0.1	0.23	0.97	0.98	
35	-	unknown		22.2	0.2 ± 0	0.1 ± 0	-0.46	-0.89	0.56	
36	222	sesquiterpene		22.4	0 ± 0	0.1 ± 0	0.99	-0.13	0.13	
37	222	sesquiterpene		22.5	0 ± 0	0.3 ± 0.3	0.99	0.12	0.05	*
38	222	sesquiterpene		23.1	0 ± 0	0.2 ± 0.2	0.99	0.13	0.01	**
39	264	terpenoid		24.3	0 ± 0.1	0 ± 0	-0.18	0.98	0.01	**
40	264	terpenoid		24.2	0.1 ± 0.1	0.1 ± 0	0.15	-0.99	0.34	
41	264	terpenoid		25.1	0.3 ± 0.2	0.1 ± 0.1	-0.53	0.85	0.19	
42	-	unknown		26.1	0.1 ± 0.1	0 ± 0	-0.27	-0.96	0.19	
43	-	unknown		26.3	0.1 ± 0.1	0 ± 0	-0.30	-0.95	0.12	
44	-	unknown		29.4	0.1 ± 0.1	0.1 ± 0.1	-0.42	0.91	0.11	
45	-	unknown		29.7	4.1 ± 2.7	4.1 ± 3.3	-0.43	0.90	0.30	
46	-	unknown		30.0	3.1 ± 0.8	1 ± 1.2	-0.73	-0.69	0.01	**
47	-	unknown		30.2	0.2 ± 0.1	0.1 ± 0.1	-0.35	-0.94	0.01	**
48	-	unknown		32.1	0.2 ± 0.1	0 ± 0	-0.43	-0.90	0.08	.
49	-	unknown		32.4	0.1 ± 0.1	0 ± 0.1	-0.36	0.93	0.04	*
50	-	unknown		32.6	0 ± 0	0.1 ± 0	0.94	0.35	0.51	
51	-	diterpene		34.5	0.2 ± 0	0.4 ± 0.2	0.98	0.20	0.04	*
52	-	diterpene		34.7	0.3 ± 0	0.7 ± 0.3	0.97	0.23	0.06	.
53	-	unknown		35.8	0.2 ± 0.1	0 ± 0.1	-0.69	0.72	0.13	
54	-	terpenoid/steroid		35.9	0.7 ± 0.3	1.1 ± 0.4	0.50	0.87	0.16	
55	-	terpenoid/steroid		36.0	0.2 ± 0.1	0 ± 0	-0.61	0.79	0.11	
56	-	terpenoid/steroid		36.2	1.2 ± 0.2	0.8 ± 0.1	-0.92	0.40	0.30	
57	-	terpenoid/steroid		36.4	0.2 ± 0.1	0 ± 0	-0.50	0.87	0.14	
58	-	terpenoid/steroid		36.4	1.4 ± 0.2	1.2 ± 0.1	-0.78	0.62	0.22	
59	-	terpenoid/steroid		36.5	1.4 ± 0.1	1.1 ± 0.1	-0.91	-0.41	0.28	
60	-	unknown		36.6	0.2 ± 0.1	0 ± 0	-0.99	-0.14	0.57	
61	-	terpenoid/steroid		36.6	0.9 ± 0.4	0.5 ± 0.1	-0.37	0.93	0.11	
62	-	unknown		36.9	0.5 ± 0.7	0 ± 0	-0.24	0.97	0.22	
63	-	unknown		37.2	4.8 ± 2.9	2.7 ± 0.8	-0.16	0.99	0.24	
64	-	terpenoid/steroid		37.3	8.7 ± 3.6	9.4 ± 1.1	-0.02	-1.00	0.04	*
65	-	terpenoid/steroid		37.6	8.7 ± 1.1	7.8 ± 0.2	-0.25	-0.97	0.08	.
66	-	terpenoid/steroid		37.9	0 ± 0	0.1 ± 0	0.77	-0.64	0.56	
67	-	terpenoid/steroid		38.0	0.1 ± 0	0.1 ± 0	-0.19	0.98	0.72	
68	-	unknown		38.1	0.3 ± 0.3	0.1 ± 0.2	-0.44	0.90	0.13	
69	-	unknown		38.1	0.2 ± 0.3	0.6 ± 0.4	0.80	-0.60	0.19	
70	-	unknown		38.3	0.1 ± 0.1	0.1 ± 0.1	-0.37	0.93	0.09	.
71	-	unknown		38.4	0.2 ± 0	0.4 ± 0.2	0.97	0.24	0.16	
72	-	unknown		38.5	0.2 ± 0.1	0.3 ± 0.2	0.51	0.86	0.24	
73	-	unknown		38.8	0.8 ± 0.2	0.9 ± 0.3	-0.39	0.92	0.93	
74	-	terpenoid/steroid		38.8	0.1 ± 0.1	0.1 ± 0	0.03	-1.00	0.92	
75	-	terpenoid/steroid		39.0	0 ± 0	0.1 ± 0.1	0.83	0.56	0.14	
76	-	unknown		39.1	0 ± 0	0.4 ± 0.4	0.98	0.20	0.15	
77	-	unknown		39.3	11.4 ± 1.2	4.3 ± 4.2	-0.97	0.25	0.02	*
78	-	unknown		39.4	0 ± 0	1.2 ± 1.3	0.98	0.19	0.15	
79	-	unknown		39.4	4.7 ± 0.2	2.1 ± 2.7	-1.00	0.02	0.10	.
80	-	unknown		39.7	1 ± 0.2	0.4 ± 0.4	-0.86	0.51	0.02	*
81	-	terpenoid/steroid		39.7	0.1 ± 0.1	0.1 ± 0.1	-0.29	-0.96	0.37	
82	-	terpenoid/steroid		40.1	0.1 ± 0.1	0 ± 0.1	-0.72	0.69	0.07	.
83	-	unknown		40.9	0.1 ± 0	0.6 ± 0.5	0.99	0.17	0.08	.
84	-	triterpene		41.6	0.1 ± 0	0.2 ± 0.1	0.82	0.57	0.09	.
85	-	triterpene		41.8	0.8 ± 0.3	2.3 ± 1.2	0.91	0.41	0.09	.
86	-	triterpene		42.1	0.1 ± 0.1	0.1 ± 0	-0.10	-1.00	0.77	
87	-	triterpene		42.2	0.1 ± 0	0 ± 0	-0.95	-0.32	0.08	.
88	-	triterpene		42.6	0.2 ± 0	0.3 ± 0.1	1.00	0.00	0.07	.
89	-	unknown		42.8	0.2 ± 0.1	0.2 ± 0.1	-0.58	0.81	0.09	.
90	-	triterpene		42.9	0.3 ± 0	0.1 ± 0.1	-0.95	-0.31	0.06	.
91	-	triterpene		42.9	0.1 ± 0.2	0.3 ± 0.3	0.55	0.83	0.03	*
92	-	triterpene		43.0	0.1 ± 0.1	0.1 ± 0.2	-0.41	-0.91	0.11	
93	-	triterpene		43.1	0 ± 0	0.1 ± 0.1	0.71	0.70	0.01	**
94	-	unknown		43.6	1.2 ± 0.2	0.5 ± 0.4	-0.91	0.40	0.02	*
95	-	Triterpene		45.7	0 ± 0	0.4 ± 0.3	0.99	0.14	0.01	**
96	-	Unknown		47.4	0 ± 0	1.6 ± 2.8	0.97	0.26	0.17	
97	-	Unknown		47.5	0 ± 0	0.6 ± 1	0.97	0.26	0.17	

## Discussion

Hybridisation between plants may result in novel genotypes, and/or may increase genetic variation, and/or may result in fixed heterosis thus giving hybrids adaptive advantages that enable them to become invasive [4]. Our study shows that hybrids can inherit some of the characteristics of *C*. *torelliana* that theoretically enable them to be dispersed by bees. Some fruits inherited characteristics such as column collapse, copious quantities of resin and similar fruit dimensions to *C*. *torelliana*. Resin chemistry in one out of three hybrids examined was also quite similar to *C*. *torelliana*. However, due to the large variation in resin chemistry between hybrids and the limited sample size of only three trees analysed, conclusions with regard to resin chemistry need to be confirmed by additional analyses.

Fruit features such as size, hollowness, resin production and seed size were variable in hybrids compared to *C*. *torelliana* and *C*. *citriodora*. Fruit size is a crucial characteristic of *C*. *torelliana* that enables bee dispersal as bees can only gain entry if the fruit is large enough [8]. Hybrid fruits were on average intermediate in size between the parent species, but there was variability in the hybrids and some trees had fruits that were similar in size to *C*. *torelliana*. Those with similar fruit dimensions to *C*. *torelliana* are the most likely to allow bees to enter, and could potentially be dispersed by bees.

If bees can gain entry to the fruits they also need to be able to manoeuvre inside the fruit to collect resin [8, 10]. The *C*. *torelliana* fruit is hollow and this enables bees to manoeuvre. The hollowness is caused by the collapse of the central column and is to date has only been reported for *C*. *torelliana*. In this study four hybrids trees also showed complete column collapse, similar to *C*. *torelliana*. This indicates that this capsule characteristic is inherited by some hybrids and would facilitate bee entry into the capsule.

In other plants that are dispersed by bees, the bees collect the mucilaginous sticky exocarp and seeds [32], or collect resin and seeds from the exposed pod [33]. *C*. *torelliana* presents copious quantities of resin inside the fruits and this is a crucial adaptation to seed dispersal by stingless bees, because it attracts the bees and allows the seeds to attach to the bees’ corbiculae [8]. All hybrid fruits contained some resin, and one hybrid even showed similar resin quantities to *C*. *torelliana*. Interestingly bees foraged on hybrid fruits only when they had been damaged by cockatoos and the resin had been exposed, suggesting that bees were not able to access the hybrid resins in the fruits when they were intact. Clearly the resin is attractive to the bees, but the hybrid fruit structure may have prevented bees from detecting the resin and thus foraging on it.

Seed size of hybrids was noticeably larger than *C*. *torelliana* in some hybrids (Fig 3C). Bees can transport up to 4 *C*. *torelliana* seeds at one time so the larger hybrid seeds are unlikely to prevent seed transport completely, although the larger hybrid seeds are likely to be harder to transport by bees [9].

*C*. *torelliana* resin can easily be distinguished from resins of other tree species. It consists of a variety of terpenoids, phloroglucinols and flavonoids, with minor differences between trunk resin and fruit resin [34, 35]. Stingless bees accurately learn its volatile profile as minor changes to the profile strongly reduce the attraction of bees [22]. This high cue specificity shown by bees indicates that *C*. *torelliana* fruit resin chemistry is highly species-specific.

We found that one hybrid had a chemical profile that was very similar to *C*. *torelliana*, whereas the two others differed in the proportions of some compounds or were even entirely lacking compounds typically found in *C*. *torelliana* resin. Consequently, the resin of hybrids can strongly resemble *C*. *torelliana* resin, particularly with regard to volatile compounds. Because stingless bees use a blend of volatile mono- and sesquiterpenes to find resin sources and are very specific in their olfactory search ‘image’ when foraging on *C*. *torelliana* fruit resin [22, 24], they may be as strongly attracted to the hybrid resin as to *C*. *torelliana* resin, unless morphological fruit characteristics prevent them from actually smelling the resin. However, this assumption needs to be confirmed by additional analyses of hybrid resin samples.

Some hybrid fruits showed the critical features of *C*. *torelliana* fruit structure that are essential for long distance dispersal by bees. However, we did not actually observe bees foraging on intact hybrid fruits of *C*. *torelliana* in this study. Moreover, we did not find a hybrid with the complete set of characters that would enable seed dispersal. This suggests that fruit characteristics that enable bee dispersal are not inherited together and are probably under the control of many genes. Many traits of eucalypts are inherited in a more-or-less intermediate manner in F1 hybrids, although there are exceptions, and dominance or partial dominance of parental traits have sometimes been reported [36]. For example, leaf chemistry of *C*. *torelliana* hybrids shows complex heritability patterns. Hybrids between *C*. *torelliana* and *C*. *citriodora* subsp. *variegata* show intermediate chemical leaf profiles, or contain compounds from both parents [37, 38]. In contrast, the foliar chemistry of hybrids between *C*. *torelliana* and *C*. *citriodora* subsp. *citriodora* and *C*. *henryi* was always dominated by the *C*. *torelliana* parent.

Although bee dispersal of hybrids was not observed in this study, we examined only hybrids between *C*. *torelliana* and *C*. *citriodora* subsp. *citriodora*. Further study on other hybrids is needed to more completely assess the risk of bee dispersal. The risk of seed dispersal by bees in hybrids will depend on how frequently, and in what combinations the *C*. *torelliana* fruit characters occur in the hybrid population. Spotted gums may inherit some of the *C*. *torelliana* fruit characteristics from hybrids as hybrids can successfully backcross onto the parents [16]. Around 20,000 hybrids have been planted in Australia [18] and large scale hybridisation programs are still underway. These hybrids include species not examined in this study such as *C*. *citriodora* subsp. *variegata* and *C*. *henryi*. It is likely that some of these hybrids inherit the adaptive advantages such as long distance seed dispersal that enable them to become invasive [4]. We therefore need ongoing monitoring of fruit characteristics in these hybrids to minimize the chance of long distance seed dispersal and invasion by these hybrid plantation trees.
